# Association of pancreatitis with risk of diabetes: analysis of real-world data

**DOI:** 10.3389/fcdhc.2023.1326239

**Published:** 2024-01-09

**Authors:** Djibril M. Ba, Vernon M. Chinchilli, Anna M. Cozzi, David P. Bradley, Ariana R. Pichardo-Lowden

**Affiliations:** ^1^ Department of Public Health Sciences, Penn State College of Medicine, Hershey, PA, United States; ^2^ Department of Medicine, Division of Endocrinology, Diabetes and Metabolism, Penn State Health Milton S. Hershey Medical Center, Hershey, PA, United States

**Keywords:** diabetes, pancreatitis, type 3 diabetes, database analysis, MarketScan Research Data, diabetes incidence, pancreatitis complication

## Abstract

**Introduction:**

Diabetes is a major cause of disease burden with considerable public health significance. While the pancreas plays a significant role in glucose homeostasis, the association between pancreatitis and new onset diabetes is not well understood. The purpose of this study was to examine that association using large real-world data.

**Materials and methods:**

Utilizing the IBM^®^ MarketScan^®^ commercial claims database from 2016 to 2019, pancreatitis and diabetes regardless of diagnostic category, were identified using *International Classification of Diseases, Tenth Revision* [*ICD-10*] codes. We then performed descriptive analyses characterizing non-pancreatitis (NP), acute pancreatitis (AP), and chronic pancreatitis (CP) cohort subjects. Stratified Cox proportional hazards regression models were used to estimate hazard ratios (HRs) and 95% confidence intervals (CI) of diabetes across the three clinical categories.

**Results:**

In total, 310,962 individuals were included in the analysis. During 503,274 person‐years of follow‐up, we identified 15,951 incident diabetes cases. While men and women had higher incidence rates of CP and AP-related diabetes, the rates were significantly greater in men and highest among individuals with CP (91.6 per 1000 persons-years (PY)) followed by AP (75.9 per 1000-PY) as compared to those with NP (27.8 per 1000-PY). After adjustment for diabetes risk factors, relative to the NP group, the HR for future diabetes was 2.59 (95% CI: 2.45-2.74) (P<0.001) for the CP group, and 2.39 (95% CI: 2.30-2.48) (P<0.001) for the AP group.

**Conclusion:**

Pancreatitis was associated with a high risk of diabetes independent of demographic, lifestyle, and comorbid conditions.

## Introduction

Pancreatic diseases, including acute pancreatitis (AP) and chronic pancreatitis (CP) constitute a very challenging medical disorder, and their relationship with the risk of developing diabetes is uncertain. AP, an inflammatory disease arising from the exocrine pancreas, is the single most frequent gastrointestinal disorder (1), causing more than 300,000 hospitalizations per year, with healthcare costs exceeding $2 billion annually in the United States (US) (2, 3). According to a recent report from the centers for disease control and prevention (CDC), in 2019, diabetes affects approximately 37.3 million Americans, or 11.3% of the US population) (4). Diabetes is the eighth leading cause of death in the US, and the risk of death among individuals with diabetes is almost double that of people without diabetes (5). From an economic perspective, diabetes constitutes a substantial financial burden in the US, with an estimated total direct and indirect cost of 327 billion in 2017 (6). Diabetes secondary to pancreatic disease (post-pancreatitis diabetes, or a form of type “3c” diabetes) results primarily from structural and functional loss of glucose-normalizing insulin production and is frequently either misdiagnosed as type 2 diabetes or underdiagnosed (7). It is likely the most common complication after AP or CP, with a cumulative incidence ranging from 23% to 40% (8–10). Although hyperglycemia is a common feature in patients with AP or CP, the likelihood of developing diabetes, and its temporal and prognostic course after diagnosis, is not well-described (11, 12). According to two recent meta-analyses, the incidence rate of diabetes secondary to AP indicates that about 23% of AP patients will develop diabetes within three years of discharge (8, 9). However, the studies included are limited by small sample sizes and are confounded by the frequent occurrence of stress hyperglycemia. Stress hyperglycemia is a transient elevation of glucose levels in the context of acute illness that may signal progression to prediabetes and type 2 diabetes (13, 14), which can also be anticipated from admission glucose level (15). This may represent a confounder to the diagnosis of AP-related incident diabetes. In addition, whether traditional risk factors for diabetes (overweight/obese status, prediabetes, family history, older age, history of gestational diabetes, presence of non-alcoholic fatty liver disease (NAFLD), smoking status, etc.) also play a role in diabetes development, in the context of CP or AP is largely unknown.

Therefore, we sought to examine the association of pancreatitis (AP and CP) with new-onset diabetes while assessing potential explanatory factors that may contribute to diabetes development. We employed the largest cohort of patients studied to date using real-world data from the IBM^®^ MarketScan^®^ commercial claims database from 2016 to 2019.

## Materials and methods

### Data source

We conducted a retrospective cohort study using the IBM^®^ Watson Health MarketScan^®^ Commercial Claims and Encounters Research Databases from 2016 to 2019. The study period was selected based on the transition from the International Classification of Diseases, Ninth Revision, Clinical Modification (ICD-9) to the 10^th^ revision, which occurred in late 2015. This avoids potential misclassification bias by including two different iterations of the classification system. The MarketScan^®^ is a large nationwide database that consists of reimbursed health care claims for employees, their spouses, and dependents of over 300 employers and health plans across 50 US states and the District of Columbia. The database includes claims information from more than 130 payers and describes the health care service use for more than 40 million covered employees and family members per year (16). The MarketScan^®^ databases have been widely used in large epidemiologic outcomes research and health economic studies (17, 18), and is divided into subsections, including inpatient claims, outpatient claims, outpatient prescription drug claims, and enrollment details information. The MarketScan^®^ databases provide a comprehensive data set that is strong longitudinally, detailed at the patient level and reflects the continuum of care. The study was deemed to be exempt from Institutional Review Board evaluation.

### Study population

The study sample included adults 18 to 64 years of age who had at least one inpatient or outpatient medical claim for diagnosed pancreatitis using *ICD-10* codes. Recognized *ICD-10* codes included K85 for AP, B25.2 for cytomegalovirus [CMV] pancreatitis, and K86.0, K86.1, K86.89 for CP. We combined CMV pancreatitis with AP because of the small number of CMV-related diseases and reported it as one category of AP. We excluded individuals diagnosed with pancreatic cancer (*ICD-10* code C25.9) during the study period and/or a diagnosis of diabetes (*ICD-10* codes: E10 for type 1 diabetes, E11 for type 2 diabetes, E13 for indeterminate diabetes, and O24.4, O24.8, O24.9 for gestational diabetes) prior to the index date, defined as the date of first pancreatitis diagnosis for all study participants.

There were a total of 71,245 participants with pancreatitis (cases) meeting the inclusion and exclusion criteria. Each individual with pancreatitis was randomly matched by gender and age (year of birth) with up to four individuals from the general population (1:4) without pancreatitis (NP) (n=239,717), as illustrated in **Supplementary Figure 1**. These NP subjects had been hospitalized for other causes. None of the individuals included in this study had an *ICD-10* code for diabetes at the index date (baseline). When higher numbers of NP individuals were available, a maximum of four were chosen at random. The index date for AP and CP individuals was also assigned to the matched controls without pancreatitis (NP).

### Assessment of outcome

The primary outcome of interest was the incidence of diabetes, defined by *ICD-10* codes occurring during the follow-up period. The average time between the index date of pancreatitis and diagnosis of diabetes was ~1 year. Diabetes diagnoses were identified by the presence of *ICD-10* codes E11 and E10. We also included *ICD-10* code E13 if one of the following *ICD-10* diagnoses were present during follow-up: gestational diabetes mellitus or diabetes in pregnancy O24.4, O24.8, and O24.9.

### Assessment of covariates

Data on age (years), sex (men/women), and US census region (South, West, North Central, Northeast) were collected from the database. Based on a comprehensive literature review, the following potential confounders, representing risk factors for diabetes development, were also captured using their corresponding *ICD-10*, or Current Procedural Terminology (CPT) codes (**Supplementary Table 1**): overweight or obesity, prediabetes, family history of diabetes, personal history of gestational diabetes, non-alcoholic fatty liver disease (NAFLD), alcohol use, tobacco use, and hypertension. The 12 months preceding the index date of pancreatitis were established as the baseline period.

### Statistical analysis

Descriptive analyses across three clinical categories (NP, AP, and CP) were performed to describe the characteristics of the study cohort. The person-years for each participant was accumulated from the index date to the first occurrence of an outcome of interest, end of enrollment, or end of the study period (December 31, 2019), whichever occurred first. The proportional hazards assumption was violated (*P*<0.001), which could be because of the large sample size of the current study. Thus, a stratified Cox proportional hazards regression analysis was performed to calculate hazards ratios (HRs) and corresponding 95% CIs for the risk of developing diabetes across the three clinical categories, with NP as the reference group, controlling for age, sex, region, overweight or obese, prediabetes, family history of diabetes, personal history of gestational diabetes, NAFLD, alcohol use, tobacco use, and hypertension.

We conducted a subgroup secondary analyses by calculating the unadjusted incidence rates and corresponding 95% CI per 1000 person-years of diabetes according to the three groups of pancreatitis status (AP, CP, or NP). Data were analyzed in SAS Software version 9.4 (SAS Institute Inc; Cary, NC) at a two-tailed alpha level of 0.05.

## Results

In total, 310,962 individuals (mean [SD] age 46.1 [12.4] years) were included in the final analysis. During 503,274 person‐years of follow‐up, we identified 15,951 incident diabetes cases. Out of the compiled cases, 46% (n=141,712) were male and 54% (n=169,220) were female, with a female preponderance in all three cohorts (54.4% of NP, 53.5% of AP, and 58.5% of CP cases). **Table 1** denotes the descriptive report of matched covariates (age and gender) and other potential confounders by pancreatitis status. Compared with control subjects (NP), individuals with AP had higher proportions of alcohol use and overweight/obesity, and were mostly from the South region of the US. Additionally, individuals with CP exhibited higher proportions of tobacco use, prediabetes, NAFLD, and hypertension (**Table 1**). Of note, individuals with NP had the highest prevalence of personal history of gestational diabetes (**Table 1**).

**Table 1:** Baseline basic characteristics according to pancreatitis status (n=310,962).

Characteristics	No Pancreatitis n=239,717	Acute Pancreatitis n=55,220	Chronic Pancreatitis n=16,025	P value
Age, mean (SD), y	46.2 ± 12.4	45.0 ± 12.5	49.2 ± 11.5	
sex, %				
Men	109,397 (45.6)	25,696 (46.5)	6,649 (41.5)	
Women	130,320 (54.4)	29,524 (53.5)	9,376 (58.5)	
Region of US, %				
Northeast	40,726 (17.0)	8,399 (15.2)	3,039 (19.0)	
North Central	59,843 (25.0)	11,405 (20.7)	3,424 (21.4)	<.0001
South	101,311 (42.3)	27,103 (49.1)	7,105 (44.3)	
West	37,837 (15.8)	8,313 (15.1)	2,457 (15.3)	
Tobacco use, %	6,317 (2.6)	4,256 (7.7)	1,306 (8.2)	<.0001
Alcohol use, %	1,570 (0.7)	2,881 (5.2)	588 (3.7)	<.0001
Overweight/obesity, %	14,643 (6.1)	5,822 (10.5)	1,505 (9.4)	<.0001
Prediabetes, %	784 (0.3)	193 (0.4)	126 (0.8)	<.0001
Family history of diabetes, %	247 (0.1)	50 (0.1)	11 (0.1)	<.31
NAFLD, %	1,974 (0.8)	2,825 (5.1)	1,129 (7.1)	<.0001
Hypertension, %	34,944 (14.6)	12,958 (24.3)	3,887 (24.3)	<.0001
Personal history of gestational diabetes, %	77 (0.03)	12 (0.02)	4 (0.02)	.41

*NAFLD, non-alcoholic fatty liver disease.

Among men, the unadjusted incidence rate of diabetes was higher among individuals with CP (91.6 per 1000 persons-years [PY]), followed by AP (75.9 per 1000-PY) and NP (27.8 per 1000-PY). Among women, the unadjusted incidence rates of diabetes were also higher among individuals with CP and AP as compared to those with NP, but significantly lower than men. For all three groups, we observed a linear dose-response relationship between advancing age and unadjusted incidence rates of diabetes. Among the age group 55-64 years, individuals with CP had the highest unadjusted incidence rate of diabetes (85.2 per 1000-PY) followed by those with AP (78.6 per 100-PY), and individuals with NP (40.0 per 1000-PY).

We next examined several canonical risk factors conferring a higher risk of DM in the general population (19) (**Table 2**). Individuals with alcohol use and AP had a higher unadjusted incidence rate of diabetes compared to those who did not use alcohol; this rate was lower than the unadjusted incidence rate of diabetes in both corresponding CP groups. However, the unadjusted incidence rate of diabetes in individuals with CP and alcohol use, as compared to those who did not use alcohol, was significantly lower. In contrast, individuals with tobacco use and CP or AP unexpectedly had a lower unadjusted diabetes incidence compared to those who did not use tobacco. Similar to alcohol use, among those categorized as overweight or obese, the unadjusted incidence rate of diabetes was lower for individuals with CP compared to those who were not overweight or obese, indicating a potential disassociation between weight status and diabetes in the CP group.

**Table 2:** Subgroups analysis results showing the unadjusted incidence rates, 95% CI per 1000 person-years of diabetes according to the three groups of pancreatitis status.

Characteristics	No Pancreatitis	Acute Pancreatitis	Chronic Pancreatitis
Sex			
Men	27.8 (27.0, 28.6)	75.9 (73.0, 78.9)	91.6 (85.2, 98.4)
Women	21.3 (20.7, 21.9)	48.6 (46.6, 50.8)	62.7 (58.2, 67.4)
Age groups, y			
18-34	5.9 (5.4, 6.5)	28.4 (25.9, 31.0)	44.6 (37.1, 53.5)
35-44	15.2 (14.4, 16.1)	55.1 (51.6, 58.8)	71.1 (62.6, 81.0)
45-54	27.1 (26.2, 28.1)	72.9 (69.4, 76.6)	76.2 (70.0, 83.5)
55-64	40.0 (38.9, 41.2)	78.6 (74.9, 82.5)	85.2 (78.9, 92.1)
Region of US, %			
Northeast	23.2 (22.1, 24.4)	57.9 (53.8, 62.2)	70.6 (62.8, 79.4)
North Central	22.8 (21.9, 23.7)	58.7 (55.0, 62.6)	64.7 (57.5, 72.8)
South	26.8 (26.0, 27.6)	66.0 (63.4, 68.7)	82.8 (76.8, 89.1)
West	20.8 (19.7, 22.0)	51.0 (46.9, 55.4)	71.5 (62.3, 82.2)
Tobacco use			
No	24.2 (23.7, 24.7)	61.4 (60.0, 63.3)	76.7 (72.8, 80.9)
Yes	26.9 (23.7, 30.5)	53.8 (47.7, 60.6)	50.8 (40.6, 63.6)
Alcohol use			
No	24.3 (23.8, 24.8)	60.6 (58.8, 62.4)	74.8 (71.0, 78.8)
Yes	22.2 (16.8, 29.3)	67.6 (59.2, 77.2)	71.7 (53.9, 95.4)
Overweight/obesity			
No	23.5 (23.0, 24.0)	60.3 (58.5, 62.2)	75.2 (71.2, 79.3)
Yes	38.4 (35.8, 41.2)	66.6 (61, 72.8)	69.6 (57.9, 83.7)
NAFLD			
No	24.2 (23.7, 24.6)	60.3 (58.5, 62.1)	75.4 (71.5, 79.5)
Yes	40.9 (34.0, 49.3)	73.1 (64.5, 82.7)	65.2 (52.5, 81.1)
Hypertension			
No	22.4 (21.9, 22.8)	55.4 (53.5, 57.3)	74.2 (70.0, 78.7)
Yes	37.8 (36.1, 39.5)	80.7 (76.5, 85.2)	76.4 (68.6, 85.2)
Prediabetes			
No	24.2 (23.7, 24.6)	60.7 (59.0, 62.5)	74.5 (70.8, 78.5)
Yes	89.8 (69.8, 115.4)	165.2 (110.8, 246.5)	102.5 (58.2, 180.5)

Several comorbid medical conditions are associated with higher rates of diabetes. Among those with a coexisting diagnosis of NAFLD, the highest unadjusted incidence of diabetes was found in individuals with AP (73.1 per 1000-PY; **Table 2**). In contrast, individuals with CP without NAFLD had a higher unadjusted incidence rate of diabetes than those with CP and NAFLD. The unadjusted incidence rate of diabetes was higher in individuals with hypertension in all three cohorts (NP, AP, and CP) as compared to those without hypertension, and was most pronounced in AP (80.7 per 1000-PY; **Table 2**). Across all variables, the highest unadjusted incidence rate of diabetes was among individuals with prediabetes and AP (165.2 per 1000-PY; **Table 2**), followed by CP (102.5 per 1000-PY; **Table 2**).

Overall, the unadjusted incidence rate of diabetes was remarkably higher among individuals with CP (74.7 per 1000-PY) followed by those with AP (60.9 per 1000-PY). Individuals with NP had the lowest incidence rate of diabetes (24.3 per 1000-PY) (**Table 3**).

**Table 3:** Stratified Cox Hazard ratios for the development of future diabetes among 310,962 participants when the Acute Pancreatitis group and Chronic Pancreatitis group were each individually compared with the group with no pancreatitis.

Variable	No Pancreatitis	Acute Pancreatitis	Chronic Pancreatitis
Person-years	408,493	75,359	19,422
Diabetes cases, n	9,911	4,589	1,451
Incidence rate, 95% CI per 1000 person-years	24.3 (23.8, 24.7)	60.9 (59.2, 62.7)	74.7 (71.0, 78.7)
Model 1	(reference)	2.46 (2.38, 2.55)	2.60 (2.46, 2.75)
Model 2	(reference)	2.39 (2.30, 2.48)	2.59 (2.45, 2.74)

Model 1: stratified by age (years) and sex (men/women).

Model 2: Model 1 + further stratified by US region (Northeast, North Central, South, West), tobacco use, alcohol use, overweight/obesity, prediabetes, NAFLD, hypertension (each yes vs. no).

Only significant covariates were included in the final parsimonious model.

In the age and sex-stratified Cox model (model 1), individuals with CP had a higher risk of diabetes (hazard ratio (HR) = 2.60; 95% CI: 2.46-2.75; **Table 3**) followed by the AP group (HR=2.46; 95% CI: 2.38, 2.55; **Table 3**) as compared to those with NP. Utilizing additional stratification for other potential confounding factors (model 2), the positive association between pancreatitis status and diabetes remained significant for the CP (HR = 2.59; 95% CI: 2.45-2.74; **Table 3**) and AP (HR = 2.39; 95% CI: 2.30-2.48; **Table 3**) groups, as compared to the NP group.

## Discussion

Our study examined the contribution of pancreatitis on the incidence of diabetes subsequent to either AP or CP, collectively termed post-pancreatitis diabetes mellitus (PP-DM). We accounted for important confounders, and further determined which risk factors contributed to this heightened risk. In the largest study to date, we report a significantly higher risk of DM in those individuals with either CP or AP, with CP conferring the highest risk both before and after adjustment for confounding factors. These findings are consistent with previous reports in the literature (8–10). However, the influence that other known risk factors for diabetes may concurrently exert in developing diabetes among post-pancreatitis individuals is less clear. This is clinically relevant as more proactive surveillance may be needed in certain subpopulations. While some well-known risk factors (presence of hypertension, prediabetes, advancing age, male gender) were associated with higher rates of DM in both CP and AP patients (as well as in the control group), smoking appeared to reduce the risk. In addition, in subgroup analyses, other risk factors (overweight/obese, presence of NAFLD) were related to an increased risk of AP-related DM, but not CP-related DM. These findings illustrate the heterogeneous nature of not only PP-DM, but provides insight for additional inquiry. Yet, despite this advancing knowledge and the potential benefits of early recognition, a medical history of pancreatitis is not currently included as a criterion for diabetes screening. Furthermore, persons who develop diabetes after pancreatitis may experience unique challenges related to their glycemic control and outcomes in relation to other forms of diabetes, necessitating individualized management.

The contrasting incidence of diabetes after AP and CP higher in men (75.9 and 91.6) than women (48.6 and 62.7) depicted in **Table 2**, more pronounced than in the non-pancreatitis (NP) group, is an important finding. This discrepancy was observed despite actual rates of AP and CP in men (46.5% and 41.5% respectively) being lower than in women (53.5% and 58.5% respectively) as shown in **Table 1**, suggesting overall greater risk among males. According to the United States (US) Centers for Disease Control (CDC), the estimated crude prevalence of diabetes in adults 18 and older is 15.4% (13.5–17.5, 95% CI) in men and 14.1% (11.8 and 16.7, 95% CI) in women (20). In T2DM, the male preponderance in DM rates has been multifactorial, including higher rates of obesity and sedentary lifestyle, differential adipose tissue distribution, sex hormone and adipokine/cytokine differences, and psychosocial factors, among others (21). Given our findings, further investigation in this regard may be warranted in patients with pancreatitis.

In all age groups, as shown in **Table 2**, the incidence of diabetes was higher in subjects with CP. Distinctly, among the age group 55 to 64 years, individuals with CP had the highest diabetes incidence rate followed by those with AP. The greater incidence observed in this study aligns with the estimated crude prevalence of diabetes, which is greater in older adults between 45-64 years of age [18.9% (16.1–22.1-CI)] in contrast with younger groups in the US, as reported by the CDC. It may therefore be anticipated that for subjects 65 years and older, not included in this study, PP-DM incidence may be even higher since the prevalence in this age group is 29.2% (26.4–32.1-CI) (20).

In our study, we performed a comprehensive subgroup analyses while accounting for known T2DM risk factors and based on no history of pancreatitis (NP), or the presence of AP or CP. Not surprisingly, across all confounding variables, the highest incidence rate of diabetes after pancreatitis was observed among individuals with prediabetes (165.2 per 1000-PY for AP and 102.5 per 1000-PY for CP). There were 1.4 million new cases of diabetes among US adults ≥ 18 years in the United States in 2019, highlighting the clinical relevance of prediabetes disease progression. Male gender, the presence of coexistent hypertension, and advancing age also disproportionately increased diabetes risk in AP and CP compared to NP patients. In contrast, smoking unexpectedly lessened the risk of PP-DM. Smoking is a risk factor for both AP and CP (22). In a separate analysis of our data without stratification by pancreatitis status, smoking was associated with increased risk of diabetes, consistent with other retrospective studies that included patients with pancreatitis. However, this association has not been corroborated in prospective studies. In patients without pancreatitis, the results are conflicting. In epidemiologic-based and prospective studies, smoking may or may not be associated with type 2 diabetes (23, 24). Mechanistically, smoking promotes altered body composition (i.e. increased visceral adipose tissue), insulin resistance (i.e. reduced glucose uptake) (25), reduced beta-cell function (26), and impairments in peripheral insulin signaling and free fatty acid flux (27). Therefore, our finding should be interpreted with caution, and we suspect that inaccuracies of self-reporting tobacco use in insurance claims data could introduce an error due to misclassification and possibly contribute to undercounting tobacco users using ICD-10 and CPT codes.

Other risk factors (notably overweight/obese status and presence of NAFLD) increased diabetes risk in AP, but not CP. Yet neither conferred as high a risk as the control group, indicating a possible differential effect of weight status between PP-DM and T2DM, and also potentially reflecting the nature of CP vs. AP. Our analysis did not account for the severity of AP or CP, which can impact the pancreatic parenchyma, causing detrimental effects on insulin secretion that are largely weight-independent (28). This may provide an explanation as to why overweight/obese status had limited comparative impact in CP patients. In addition, a study of 9,124 subjects with exocrine pancreatic dysfunction (EPD) due to pancreatitis had a significantly higher risk for diabetes (adjusted hazard ratio 3.83; 95% confidence interval, 2.37–6.18). The crude hazard ratio of diabetes in subjects with EPD after AP was 4.36 (2.33–8.18) and after CP was 2.2 (1.06–4.65) (29). Greater EPD in CP vs. AP may therefore present another factor influential in the development of diabetes following pancreatitis. Our study could not account for degree of EPD, or for severity of pancreatitis or state of dysglycemia during single or recurrent pancreatitis episodes. Another important consideration is the longitudinal risk of developing PP-DM after the initial insult. The incidence of diabetes after AP within and after 3 months of the incident was reported to be 60.0 and 22.5 per 1,000 person-years (30). These notions represent relevant concepts to expand upon which may lead to understanding of other factors adding to the risk and the timeline for development of diabetes. In contrast, our study explored a period of 3 years of claims data. The incidence per 1000 person-years was 60.9 and 74.7 for AP and CP respectively, which we speculate the majority was established early after the insult.

To our knowledge, this is the first large nationwide retrospective cohort study to investigate the association of pancreatitis and diabetes using real-world data among the privately insured population. while simultaneously considering potential confounding factors. However, this study has important limitations. This is an observational study that used claims data and therefore causality cannot be inferred. The study analyzed only commercially insured individuals. Therefore, the results may only be generalizable to a similar study population, particularly since approximately 8.3% of the population are uninsured (31). The cohort included individuals under 64 years of age only, thus potentially removing the influence of older subjects whose prevalence of diabetes is expected to be higher. The study did not analyze the association of PP-DM based on the presumed etiology corresponding to their respective ICD-10 codes, and therefore does not attempt to draw conclusions related to pathophysiologic mechanisms. Also, it is known that prediabetes and diabetes unawareness is quite prevalent and we anticipate that some subjects in this cohort may have had established diabetes, or were at risk for diabetes, by the time the diagnosis was documented and an *ICD-10* code entered. Finally, some critical covariates, such as racial/ethnic disparities, could not be considered for this study because of the lack of such information in the MarketScan^®^ database.

This study provides strong evidence regarding the occurrence of new-onset diabetes after acute or chronic pancreatitis, correcting for known predisposing factors. It also opens up new potential avenues for research into the pathophysiologic mechanisms underpinning this risk, as currently being investigated by the Consortium for the Study of Chronic Pancreatitis, Diabetes, and Pancreatic Cancer (CPDPC) (32), and the Type 1 Diabetes in Acute Pancreatitis Consortium (T1DAPC) (33), cooperative agreements jointly funded by the National Cancer Institute and the National Institute of Diabetes and Digestive and Kidney Diseases.

In conclusion, pancreatitis predisposes to diabetes development but requires more proactive monitoring. Based on the age- and sex-stratified Cox model and additional stratification for other potential confounding factors, our study found that the risk of diabetes is more than double in individuals with pancreatitis. Current practice guidelines outline criteria for screening for diabetes or prediabetes in asymptomatic adults (7). Considering the attribution of risk, it is advisable that practice recommendations consider pancreatitis as a criterion for diabetes screening. Appropriate and early screening could result in preventive measures and more proactive management of diabetes.

## Data availability statement

The data analyzed in this study was obtained from Merative Marketscan Research Databases through a license from Penn State. Requests to access these datasets should be directed to Merative Marketscan.

## Ethics statement

The requirement of ethical approval was waived by Penn State Institutional Review Board for the studies on humans because the study was deemed to be exempt from Institutional Review Board evaluation. The studies were conducted in accordance with the local legislation and institutional requirements. Written informed consent for participation was not required from the participants or the participants’ legal guardians/next of kin in accordance with the national legislation and institutional requirements. The human samples used in this study were acquired from Study was conducted using the IBM^®^ Watson Health MarketScan^®^ Commercial Claims and Encounters Research Databases.

## Author contributions

DMB: Conceptualization, Supervision, Visualization, Writing – original draft, Writing – review & editing, Investigation, Project administration, Resources, Data curation, Formal Analysis, Software, Validation. VC: Conceptualization, Writing – review & editing, Resources, Supervision, Funding acquisition, Methodology. AC: Writing – review & editing. DPB: Conceptualization, Supervision, Writing – original draft, Writing – review & editing, Validation. AP-L: Writing – review & editing, Conceptualization, Investigation, Methodology, Project administration, Resources, Supervision, Validation, Visualization, Writing – original draft.
