# Supplementary material for: Association of pancreatitis with risk of diabetes: analysis of real-world data
Source: Front Clin Diabetes Healthc. 2024 Jan 9;4:1326239. doi: 10.3389/fcdhc.2023.1326239 (PMC10803589; doi:10.3389/fcdhc.2023.1326239)
Supplement: Supplementary file 1 [file Table_1.docx]

**Supplementary Table 1:** Diagnosis and procedure codes to assist in baseline medical conditions, and pre-existing conditions identification.

| **Variables** | **ICD-10 codes** | **CPT/HCPCS codes** |  |
| --- | --- | --- | --- |
| Overweight & obesity | E66 |  |  |
| Tobacco use | F17, Z71.6, Z72.0, Z87.891, O99.33, Z87.891, T65.2 | 99406, 99407, G0375, G0376, G0436, G0437, G9016, G9276, G9458, G8402, G8403, G8453, G8454, S4990, S4991, S4995, S9075, S9453, 4000F or 4001F |  |
| Alcohol use | F10, G31.2, G62.1, G72.1, I42.6, K29.2, K70, K85.2, K86.0, T51, Y91.2, Y91.3 |  |  |
| NALD | K74.60, K76.0, K76.89, K76.9, E88.89 |  |  |
| Hypertension | I10-I13, I15 |  |  |
| Prediabetes | R73.03 |  |  |
| Family History of Diabetes | Z83.3 |  |  |
| Personal history of gestational diabetes | Z86.32 |  |  |

*NALD: Non-alcoholic fatty liver disease
