# Supplementary figures and images for: Association of pancreatitis with risk of diabetes: analysis of real-world data
Source: Front Clin Diabetes Healthc. 2024 Jan 9;4:1326239. doi: 10.3389/fcdhc.2023.1326239 (PMC10803589; doi:10.3389/fcdhc.2023.1326239)

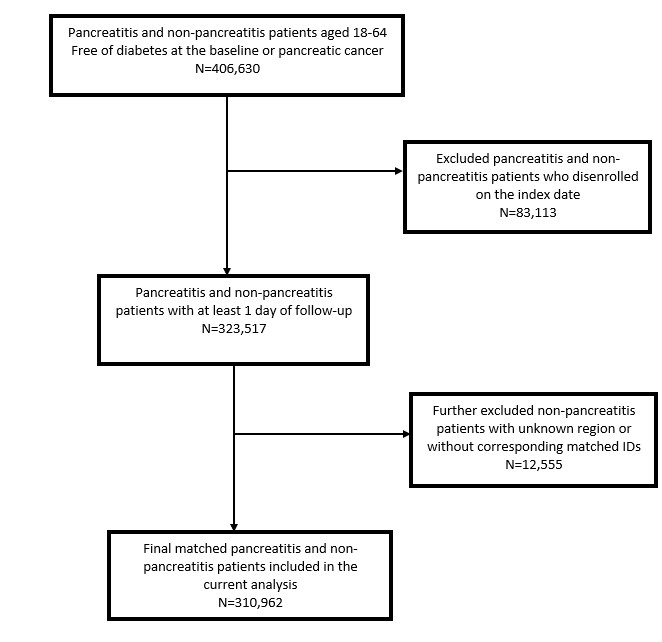

Supplement: Supplementary file 2 [file Image_1.jpeg]
